# Ageing and gastrointestinal sensory function: altered colonic mechanosensory and chemosensory function in the aged mouse

**DOI:** 10.1113/JP271403

**Published:** 2016-01-19

**Authors:** Christopher Keating, Linda Nocchi, Yang Yu, Jemma Donovan, David Grundy

**Affiliations:** ^1^ Department of Biomedical SciencesUniversity of SheffieldSheffieldUK; ^2^ Department of Pharmacy, Pharmacology and Postgraduate MedicineUniversity of HertfordshireHatfieldHertfordshireUK

## Abstract

**Key points:**

- Remarkably little is known about how age affects the sensory signalling pathways in the gastrointestinal tract despite age‐related gastrointestinal dysfunction being a prime cause of morbidity amongst the elderly population
- High‐threshold gastrointestinal sensory nerves play a key role in signalling distressing information from the gut to the brain.
- We found that ageing is associated with attenuated high‐threshold afferent mechanosensitivity in the murine colon, and associated loss of TRPV1 channel function.
- These units have the capacity to sensitise in response to injurious events, and their loss in ageing may predispose the elderly to lower awareness of GI injury or disease.

**Abstract:**

Ageing has a profound effect upon gastrointestinal function through mechanisms that are poorly understood. Here we investigated the effect of age upon gastrointestinal sensory signalling pathways in order to address the mechanisms underlying these changes. *In vitro* mouse colonic and jejunal preparations with attached splanchnic and mesenteric nerves were used to study mechanosensory and chemosensory afferent function in 3‐, 12‐ and 24‐month‐old C57BL/6 animals. Quantitative RT‐PCR was used to investigate mRNA expression in colonic tissue and dorsal root ganglion (DRG) cells isolated from 3‐ and 24‐month animals, and immunohistochemistry was used to quantify the number of 5‐HT‐expressing enterochromaffin (EC) cells. Colonic and jejunal afferent mechanosensory function was attenuated with age and these effects appeared earlier in the colon compared to the jejunum. Colonic age‐related loss of mechanosensory function was more pronounced in high‐threshold afferents compared to low‐threshold afferents. Chemosensory function was attenuated in the 24‐month colon, affecting TRPV1 and serotonergic signalling pathways. High‐threshold mechanosensory afferent fibres and small‐diameter DRG neurons possessed lower functional TRPV1 receptor responses, which occurred without a change in TRPV1 mRNA expression. Serotonergic signalling was attenuated at 24 months, but TPH1 and TPH2 mRNA expression was elevated in colonic tissue. In conclusion, we saw an age‐associated decrease in afferent mechanosensitivity in the mouse colon affecting HT units. These units have the capacity to sensitise in response to injurious events, and their loss in ageing may predispose the elderly to lower awareness of GI injury or disease.

## Key points

- Remarkably little is known about how age affects the sensory signalling pathways in the gastrointestinal tract despite age‐related gastrointestinal dysfunction being a prime cause of morbidity amongst the elderly population
- High‐threshold gastrointestinal sensory nerves play a key role in signalling distressing information from the gut to the brain.
- We found that ageing is associated with attenuated high‐threshold afferent mechanosensitivity in the murine colon, and associated loss of TRPV1 channel function.
- These units have the capacity to sensitise in response to injurious events, and their loss in ageing may predispose the elderly to lower awareness of GI injury or disease.

**Abbreviations**
Cap: capsaicin
DRG: dorsal root ganglion
EC: enterochromaffin
GAPDH: glyceraldehyde‐3‐phosphate dehydrogenase
GI: gastrointestinal
HT: high threshold
IM: ionomycin
LSN: lumbar splanchnic nerve
LT: low threshold
OCT: optimal cutting temperature compound
qRT‐PCR: quantitative RT‐PCR
SERT: serotonin reuptake transporter
TRPV1: transient receptor potential vanilloid 1
TPH1: tryptophan hydroxylase 1
TPH2: tryptophan hydroxylase 2
WDR: wide dynamic range

## Introduction

Remarkably little is known about how age affects the sensory signalling pathways in the gastrointestinal tract, despite age‐related gastrointestinal dysfunction being a prime cause of morbidity amongst the elderly population. In humans, ageing has been shown to be associated with impaired visceral sensory perception in response to mechanical stimulation affecting both the rectum and oesophagus (Lasch *et al*. 1997; Lagier *et al*. 1999). In the human rectum, sensory thresholds for both physiological and pathophysiological rectal distensions were raised compared to healthy aged subjects (Lagier *et al*. 1999), whilst similar changes were observed in the upper gastrointestinal tract in which elderly patients reported significant decreases in pain perception upon balloon distension of the oesophagus (Lasch *et al*. 1997). Sensory impairment to nutrient stimuli has also been reported to occur with age, in which symptom scores corresponding to both nausea and pain were found to be inversely correlated with age (Gururatsakul *et al*. 2010). Ageing has also been implicated in the inability of the oesophagus to detect acid reflux (Chen *et al*. 2010) and a diminished sensory response to inflammatory evoked gastrointestinal injury (Moore & Clinch, 2004). Since nociception is a key consequence of disease or tissue injury, triggering neurogenic inflammation and pain behaviour, its attenuation with age has consequences for disease progression and seeking medical advice. The pathophysiological changes in sensory signalling processes underlying this phenomenon remain unknown, but elderly patients who exhibit reduced visceral pain perceptions are more likely to impact negatively upon health care systems in terms of delayed diagnosis, and longer and more traumatic hospital stays (Vreeburg *et al*. 1997). Age‐related changes in sensory function have been explored in other systems using animal models, which have demonstrated that selective loss of function of afferent pathways is implicated in age‐related sensory dysfunction. In peripheral sensory neurons, age‐related loss of thermal pain signalling occurred as a result of loss of Aδ‐mediated signalling processes whilst C fibre signalling was maintained, suggesting that selective loss of signalling processes may account for some of the deficits observed with age (Chakour *et al*. 1996). Furthermore molecular studies in mice have also demonstrated that in cutaneous afferent pathways, ageing affected sensory signalling processes via decreased levels of TRPV1 and Nav1.8, coupled with altered trophic support leading to reduced expression of channel proteins in peripheral afferents (Wang *et al*. 2006).

Little is known of the mechanisms that underlie impaired sensory signalling in the gastrointestinal tract, although loss of neurons does not appear to be the cause of this phenomenon, since studies on sensory signalling in the periphery have shown that age‐associated behavioural deficits on ageing do not correlate with loss of cervical and lumbar dorsal root ganglion (DRG) neurons (Bergman & Ulfhake, 1998).This contrasts with the situation in the enteric nervous system in which ageing is associated with degeneration and loss of enteric neurons (Saffrey, 2013).

We hypothesised that a loss in the functional properties of these sensory pathways contributes to impaired visceral sensory function. Afferent function in response to mechanical and chemical stimulation is highly dependent upon the expression of numerous ion channels and receptors on the nerve terminals of these neurons (Grundy, 2002). Therefore loss of function in selective ion channels expressed on the nerve terminals of visceral sensory neurons could form the basis of an age‐related loss in afferent function.

With this in mind we performed a series of experiments in which afferent recordings from segments of murine jejunum and colon were made from 3‐, 12‐ and 24‐month‐old animals in order to examine the effects of age upon gastrointestinal sensory signalling processes.

## Methods

### Ethical approval and animals

All experiments were performed in accordance with the University of Sheffield's Animal Care Committee under a UK protocol and project licence following the UK Animals (Scientific Procedures) Act 1986. Male C57BL/6J mice (Charles River, UK) aged 3, 12 and 24 months were used throughout this study. Age groupings were made in accordance with established gerontology protocols (Miller & Nadon, 2000; Flurkey *et al*. 2007). To avoid batch effects when examining the 12‐ and 24‐month animals, we were supplied with small cohorts of 3‐ and 12‐month animals alongside the 24‐month animals in order to run age matched control experiments.

All animals were housed on a 12 h light–dark cycle in a temperature‐controlled environment (20.5°C) with *ad libitum* access to standard laboratory rodent chow (2018, Harlan, Teklad), and tap water. Animals were killed using cervical dislocation, and all animal husbandry followed principles of good laboratory practice in compliance with UK laws and regulations. No postmortem examinations were carried out any animals, although all animals were given a visual inspection to look for obvious lesions and abnormalities, and all animals were monitored for signs of ill health during their housing.

### Recording of colonic and jejunal afferent nerve activity

#### Tissue preparation

A total of 81 animals were used for electrophysiological experiments at 3 months (*n* = 34), 12 months (*n* = 21) and 24 months (*n* = 26) of age. Animals were killed by cervical dislocation, and their abdominal cavity opened and bathed in cold Krebs solution (in mm: NaCl 120; KCl 5.9; NaH_2_PO_4_ 1.2; MgSO_4_ 1.2; NaHCO_3_ 25; CaCl_2_ 2.5; glucose 11.5) that was gassed with carbogen (95% O_2_, 5% CO_2_). For the colonic recording experiments, the terminal 4–5 cm of the colon and attached mesentery (containing the lumbar colonic nerves) were removed intact along with the attached neurovascular bundle containing the inferior mesenteric ganglion and the lumbar splanchnic nerve (LSN). The tissue was transferred to cold Krebs solution, and following further dissection the intact colon along with the attached nerve supply was transferred into a Sylgard‐lined organ chamber (8 ml) which was continually superfused with gassed Krebs solution at a flow rate of 8 ml min^−1^ and maintained at 33–34°C. The colonic segments were securely attached at either end to an input and outlet port. The input port was connected to a Perfusor VI syringe pump, which allowed continuous intraluminal perfusion of Krebs solution through the segments (0.2 ml min^−1^) when the outlet port was open, but allowed periodic distension when closed. Intraluminal pressure was recorded via a pressure amplifier (NL 108, Digitimer, UK) connected in series with the input port. Under a dissecting microscope, the LSNs were dissected away from the neurovascular bundle and the nerve sheath surrounding the LSN was stripped back gently in order to expose the nerve trunk. Using fine forceps, the nerve trunk was teased apart into bundles, which were then drawn into a suction electrode.

The methodology for the jejunal recordings has been described previously (Keating *et al*. 2008). Briefly, segments of jejunum 3 cm long were harvested, 10–20 cm proximal to the ileocaecal junction, such that a non‐bifurcating mesenteric bundle emanated centrally from each segment. Individual segments were placed into a Sylgard‐lined organ chamber (8 ml) as described above. The mesenteric bundle was pinned out to the base of the chamber and a mesenteric nerve was dissected out from the bundle and drawn into a suction electrode.

Both colonic and jejunal preparations were stabilised for 60 min before any experimental procedures were started. Colonic and jejunal afferent mechanosensitivity was tested via distending the intestinal segment by closing a tap attached to the outlet port. This allowed the intestinal segment to be distended to an intraluminal pressure of 60 mmHg. The tap was then opened, returning the pressure to baseline. This procedure was repeated at 1000 s intervals throughout the course of the experiment to test the reproducibility of the nerve response to ramp distensions and the response during test conditions. Drugs were added to the Krebs solution perfusing the organ bath. Nerve activity was recorded with a Neurolog headstage (NL100, Digitimer) and electrical signals amplified (NL104), filtered (NL125, band pass 200–3000 Hz) and acquired (20 kHz sampling rate) to a personal computer through a Micro 1401 MKII interface running Spike2 software (Cambridge Electronic Design, Cambridge, UK).

### Quantitative RT‐PCR

DRG neurons (T9–L2) and distal colon segments were quickly removed from 3‐ and 24‐month‐old animals and stored separately in RNAlater (Life Technologies). Total RNA was extracted using the RNeasy mini Kit (Qiagen, Valencia, CA, USA). cDNA was synthesised using a high capacity cDNA reverse transcription kit (Applied Biosystems, Carlsbad, CA, USA). Quantitative RT‐PCR (qRT‐PCR) reactions were performed in duplicate using the TaqMan gene expression master mix (Applied Biosystems), and specific TaqMan primer/probe mix (IDT) in a thermal cycler (Techne TC‐3000X, Stone, UK). A complete list of primer sequences is provided in Table 1. Genes analysed were 5‐HT_3A_ and 5‐HT_3B_ (5‐HT_3_ receptor subunits), serotonin reuptake transporter (SERT), tryptophan hydroxylase 1 (TPH1); tryptophan hydroxylase 2 (TPH2), and transient receptor potential vanilloid 1 (TRPV1). Threshold cycle (*C*_t_) values were determined for both the target and the housekeeping gene (glyceraldehyde‐3‐phosphate dehydrogenase; GAPDH), and were used to calculate Δ*C*_t_ values. Results were expressed as the relative expression (1/Δ*C*_t_) of target gene to GAPDH.

**Table 1:** **List of primers used throughout study**

				Annealing
Product	Accession number	Primer sequence (5′–3′)	Position	temperature (°C)
TRPV1	XM_006536293.2	F: AGCGAGTTCAAAGACCCAGA	539–558	60
		R: TTCTCCACCAAGAGGGTCAC	771–752	
5‐HT_3A_	(NM_013561.2)	F: CCAGTTCAAGGAGTTCAGCATA	797–818	60
		R: CCACGACCATGAGGAAGATAC	927–907	
5‐HT_3B_	NM_020274	F: GTCCTCTACTTACCACATCCG	829–849	60
		R:ATCAGGAAGATGCTAGGTATCAAG	954–931	
TPH‐1	XM_011250846.1	F: TTTCGAGTCTTTCACTGCACT	565–585	60
		R: CTAGGAGTTCATGGCAGGTG	652–633	
TPH‐2	BC120514	F: ACCATTGTGACCCTGAATCC	509–528	60
		R: GTGAGAGCATCTGTCTAACTCAG	616–594	
SERT	XM_006532301.2	F: TCACCATTATCTACTTCAGCATCT	991–1014	60
		R: AGCAGGACAGAAAGGACAATG	1090–1070	
GAPDH	XM_001476707	F: AATGGTGAAGGTCGGTGTG	55–73	60
		R: GTGGAGTCATACTGGAACATGTAG	204–181	

### Calcium imaging of dorsal root ganglion neurons

DRGs (T9–L2) from 3‐ and 24‐month‐old animals were quickly removed under a dissection microscope and transferred into cold Hanks′ balanced salt solution (HBSS; pH 7.4; Gibco Invitrogen). DRGs were initially digested with cysteine (0.7 mg ml^−1^, Sigma)‐activated papain (4 mg ml^−1^, Sigma) for 20 min at 37°C followed by incubation with 4 mg ml^−1^ collagenase (Sigma) and 4.7 mg ml^−1^ dispase (Sigma) for 20 min at 37°C. Ganglia were then washed with Dulbecco's modified Eagle's medium (DMEM)–F12 tissue culture medium (Gibco) containing 10% fetal bovine serum (Gibco) to stop the enzymatic reaction. Following this stage they were then placed in 0.5 ml tissue culture media and dissociated into single cells by mechanical trituration 10 times using a plastic Pasteur pipette. The dissociated DRG neurons were plated onto Matrigel‐coated coverslips and incubated for 24 h at 37°C.

For the calcium imaging experiments, DRG neurons were loaded with 4 μm Fura‐2 AM (Life Technologies) for 30 min at 37°C in the dark. Coverslips were then transferred to a customised bath chamber (Series 20, Warner Instruments, Hamden, CT, USA) where they were continuously superfused with Hepes buffer (containing in mm: NaCl 135; KCl 5; CaCl_2_ 2; MgCl_2_1; Hepes 10; glucose 10) for 30 min at room temperature. DRG neurons were stimulated for 3 min with capsaicin diluted into Hepes buffer and applied via gravity perfusion. Changes in intracellular calcium [Ca^2+^]_i_ were monitored in real time. Excitation lights were generated by an OptoLED light source (Cairn Research Limited, Faversham, UK) and Fura‐2 AM fluorescence was recorded using a digital camera (C4742‐95 Hamamatsu Corp., Sewickley, PA, USA), and acquired to a computer using SimplePCI software (version 6.6.0.0, Hamamatsu Corp.). [Ca^2+^]_i_ mobilisation is represented as the ratio between the fluorescence signal at 340 nm and 380 nm. Responses to capsaicin were normalised against responses to the calcium ionophore ionomycin (5 μm).

### Immunohistochemistry

Segments of colon of 2 cm from 3‐ and 24‐month‐old animals were fixed overnight at 4°C in a solution comprising 4% paraformaldehyde in 0.1 m phosphate‐ buffered saline (PBS), and then washed 3 times using PBS. The fixed tissue was cryo‐protected overnight at 4°C using 30% sucrose in PBS, and embedded in optimal cutting temperature (OCT) compound (Wolf Labs, York, UK). Using a cryostat (Bright Instrument, OTF5000, Huntingdon, UK) the OCT blocks containing the cryo‐protected fixed tissue were sectioned (10 μm thickness) onto pre‐coated glass slides (Thermo Scientific MNJ‐700).The tissues were then washed with PBS (3 × 5 min) to remove OCT and then incubated with 5% goat serum in PBS for 20 min to block non‐specific binding. This was then followed by an overnight incubation with the primary antibody at 4°C, followed by 2 h incubation with the secondary antibody at room temperature. The primary antibody used was a rabbit anti‐serotonin antibody (1:50; AbD Serotec, Kidlington, UK), and the secondary antibody was a goat anti‐rabbit secondary antibody conjugated to Cy3 (1:400; Jackson ImmunoResearch, West Grove, PA, USA). After staining the slides were washed with PBS (3 × 5 min) and were then mounted with coverslips using Vectashield mounting medium with DAPI (Vector Laboratories, Peterborough, UK). Negative controls were performed by omitting the primary antibodies. Stained sections were observed under an Olympus BX51 microscope (Tokyo, Japan). Ten random images from one specimen were acquired under 20× objective using an Olympus ColourView II digital camera for offline quantification. EC cells from young and aged samples were counted in a blinded fashion. EC cell density was expressed as cells per unit area of mucosa (measured using ImageJ 1.43u; National Institutes of Health, Bethesda, MD, USA).

### Data analysis

The mean firing frequency (impulses per second; imp s^−1^) was measured with a time constant of 10 s. Firing rates were measured using a bin width of 1 s. The stimulus–response curves (mean afferent discharge plotted against intraluminal pressure) for whole nerve or single unit activities were plotted using a customised script program (Cambridge Electronic Design, Cambridge, UK). Spontaneous nerve activity was calculated from the nerve activity recorded at the start of the distension protocol. Single unit analysis was performed off‐line using Spike2 software as previously described in order to identify single units within multiunit afferent nerve preparations (Keating *et al*. 2008). Single units assigned to an individual template were then examined using principal component analysis to identify and eliminate ambiguous waveforms.

Mechanosensitive single units were classified into high threshold (HT), wide dynamic range (WDR) and low threshold (LT) on the basis of their response profile during ramp distensions as previously described (Keating *et al*. 2008). Briefly, the magnitude of the afferent response at 20 mmHg reflecting low‐threshold (LT) activation was expressed as a percentage of the maximum response at 60 mmHg. This value is referred to as %LT and for a linear stimulus–response function would be 33%. Values higher than this reflect disproportionate low‐threshold sensitivity, while values below this have disproportionate high‐threshold sensitivity. In classifying afferent fibres, values for %LT > 55 are defined as LT. HT units are those responding with a %LT of < 15% whereas afferents with a more linear increase in discharge over the range of distension used (> 15%, < 55%) were termed WDR units.

### Statistics

All statistical analyses were performed using Prism software (version 4.00; Graphpad Software, San Diego, CA, USA). All data are expressed as means ± SEM. Data were analysed using Students *t* test, or by one‐ or two‐way ANOVA with additional *post hoc* tests performed as appropriate. A χ^2^ test was performed to determine significant differences in the prevalence of each afferent fibre type in 3‐, 12‐ and 24‐month animal. The level of significance was set at *P* < 0.05 (^*^ *P* < 0.05, ^**^ *P* < 0.01, ^***^ *P* < 0.001). *n* refers to the number of animals used in specific experimental stages of the project.

### Chemicals

5‐HT was purchased from Sigma, whilst all other drug compounds were purchased from Tocris. All salts and other chemical reagents were purchased from BDH.

## Results

### Age‐related changes in baseline afferent nerve activity in the mouse colon

**Figure 1:**
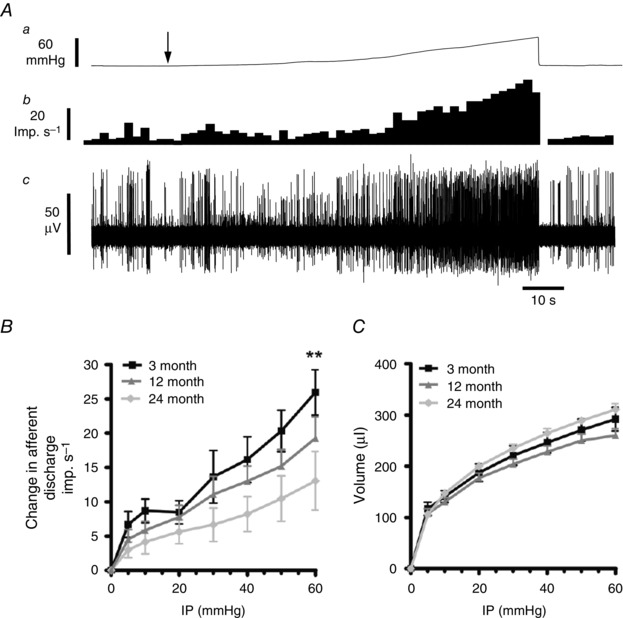
**Age‐related changes in colonic afferent mechanosensory function** *A*, ramp distension of colonic segments induced an increase in splanchnic nerve discharge in 3‐month animals. *a*, ramp distension profile. Arrow denotes start of ramp distension. *b*, nerve discharge rate. *c*, raw recording of whole nerve activity. *B*, pressure–response profiles of multiunit activity from 3‐month (*n* = 12), 12‐month (*n* = 10) and 24‐month (*n* = 13) preparations. Pressure–response profiles were attenuated at both 12 and 24 months compared to 3‐month animals. Significant differences were detected above 50 mmHg at 24 months (^**^ *P* < 0.01). *C*, the relationship between the perfusion volume and the intraluminal pressure of the colonic segment did not differ between the three ages sampled (*P* > 0.05 for 12 and 24 months *versus* 3 months).

Colonic afferent neurons exhibited a low basal afferent activity which occurred in the absence of any changes in intraluminal pressure (Fig. 1 *A*). At 3 months of age, baseline afferent discharge was 2.99 ± 0.34 imp s^−1^, which was indistinguishable from baseline activity recorded at 12 months old (2.37 ± 0.43 imp s^−1^; *P* > 0.05). At 24 months of age, baseline activity (0.98 ± 0.35 imp s^−1^) was significantly attenuated compared to 3‐month animals (*P* < 0.001).

### Age‐related changes in afferent mechanosensitivity in the mouse colon

Colonic afferent mechanosensitivity was assessed in 3‐, 12‐ and 24‐month‐old animals by making whole nerve recordings using an *in vitro* colonic‐splanchnic nerve preparation. Ramp distensions (0–60 mmHg) of colonic segments induced biphasic increases in afferent nerve discharge corresponding to the activation of low‐ and high‐threshold mechanosensitive afferent fibres (Fig. 1 *A* and *B*). In both the 12‐ and 24‐month preparations, afferent responses to ramp distensions were significantly lower than in 3‐month samples (*F* = 5.67, *P* < 0.05 and *F* = 22.8, *P* < 0.0001, respectively). We also noticed that the 24‐month afferent response to distension was significantly lower than the 12‐month samples (*F* = 7.3, *P* = 0.008). Furthermore, these changes were found to occur without any significant alterations in colonic compliance, which was unaffected at either 12 or 24 months compared to 3 months (*F *= 3.9, *P *> 0.05 and *F* = 2.3, *P* = 0.12, respectively; Fig. 1 *C*).

### The effects of age on individual mechanosensitive afferent subtypes in the mouse colon

**Figure 2:**
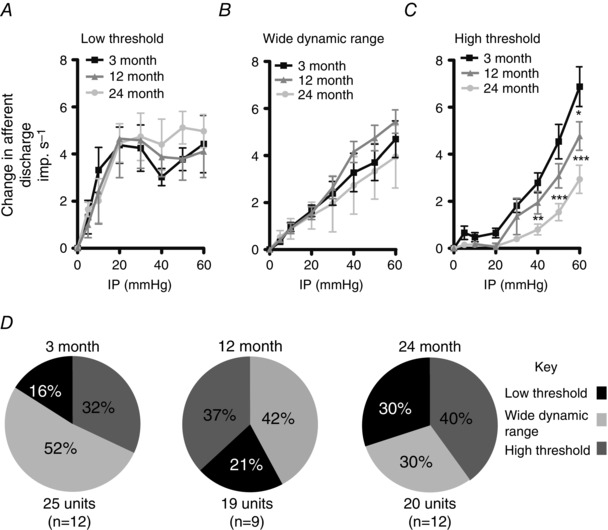
**Age‐related changes in high threshold mechanosensitive afferent function** *A*–*C*, mechanosensitive colonic afferent subtypes were identified as low threshold (*A*), wide dynamic range (*B*) and high threshold (*C*) as described in the text. The curves for both 12‐ and 24‐month high‐threshold (HT) fibres are attenuated compared to 3‐month preparations. Significant differences were seen in HT afferents at distending pressures of 60mmHg (^*^ *P* < 0.05) at 12 months, and at ≥ 40 mmHg (^**^ *P* < 0.01, ^***^ *P* < 0.001) at 24 months. The 24‐month HT afferent response profile was also significantly attenuated compared to the 12‐month preparations. Significant differences were seen at distending pressures of ≥ 50 mmHg (*P* ≤ 0.05). *D*, the relative percentages of unit subtypes identified in 3‐, 12‐ and 24‐month animals. Afferent fibre type composition is unaltered in 3‐, 12‐ and 24‐month samples. Numbers in parentheses represent the number of experiments subjected to single unit analysis.

Single unit analysis of 3‐month multiunit response profiles revealed three functional mechanosensitive afferent fibre types with distinct activation profiles (Fig. 2 *A–C*). Low‐threshold (LT) afferents were disproportionately activated by low‐pressure distensions, typically reaching a plateau of elevated firing at intraluminal pressures of approximately 20 mmHg (Fig. 2 *A*). The %LT value of LT afferents was 109.8 ± 23% (95% CI, 36.4–183.1; *n* = 4). Wide dynamic range (WDR) afferents also had a low threshold for activation, but responded in a linear fashion with afferent discharge increasing throughout the rise in intraluminal pressure suggesting that they can be activated at both physiological and nociceptive distending pressures (Fig. 2 *B*). These afferent fibre types possessed a %LT of 37.1 ± 3.3% (95% CI, 29.4–44.3; *n* = 13). The third afferent fibre types responded at higher distension thresholds and were termed high‐threshold (HT) afferents (Fig. 2 *C*). These had a %LT of 6.5 ± 1.7% (95% CI, 2.5–10.5; *n* = 8). While the majority of the HT afferents (*n* = 6) were activated at intraluminal pressures of 20 mmHg, we noted that two HT afferents were activated at intraluminal pressures of approximately 40 mmHg.

The 12‐ and 24‐month preparations also possessed LT, WDR and HT afferent fibres, but we found that age had no effect upon the relative proportions of the afferent fibre types sampled (*P* > 0.05, χ^2^ = 2.4) despite the apparent reduction in WDR fibres seen in the 12‐ and 24‐month‐old animals (Fig. 2 *D*). The 12‐month preparations possessed %LT values of 87.5 ± 25.2% (95% CI, 7.3–167; *n* = 4) for the LT afferents, 28 ± 3.5% (95% CI, 19.9–36.2; *n* = 8) and 2.7 ± 1.3% (95% CI, −1.6 to 7.1; *n* = 7) for the WDR and HT afferents, respectively. The %LT values for the 24‐month afferents were 92.5 ± 11% (95% CI, 64.2–120.8; *n* = 6) for the LT afferents, and 33.3 ± 3.7% (95% CI, 23.6–43.0; *n* = 6) and 2.6 ± 1.3% (95% CI, −0.5 to 5.7; *n* = 8) for the WDR and HT afferents, respectively. Within the 24‐month HT population we found that four afferents were activated at intraluminal pressures of 40 mmHg, and four afferents at 20 mmHg.

A comparison of the single unit afferent response profiles found that the LT and WDR afferent response profiles at 12 and 24 months were not significantly different from 3‐month responses (LT: *F* = 0.0, *P* = 0.98 and *F* = 0.78, *P* = 0.38; and WDR: *F* = 1.82, *P* = 0.17 and *F* = 0.61, *P* = 0.44, for 12 and 24 months *versus* 3 months, respectively; Fig. 2 *A* and *B*). The HT afferent response profiles (Fig. 2 *C*) at both 12 and 24 months were significantly attenuated compared to 3‐month samples (*F* = 13.06, *P* < 0.0005 and *F* = 61.4, *P* < 0.0001, respectively). Furthermore, the HT response was attenuated between 12 and 24 months of age (*F* = 13.88, *P* < 0.0003; Fig. 2 *C*).

### The effects of age on afferent chemosensitivity in the mouse colon

**Figure 3:**
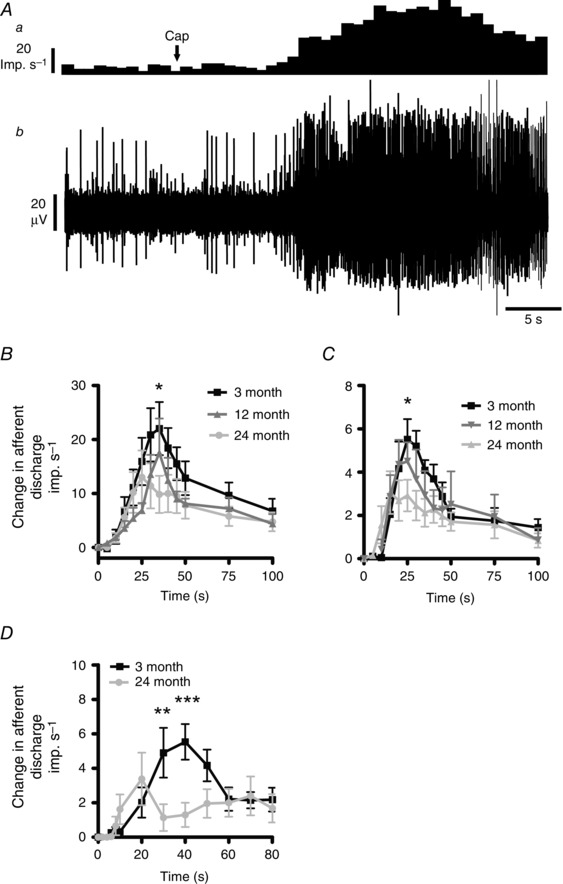
**The response of colonic splanchnic afferent nerves to capsaicin is affected by age** *A*, bath perfusion of 1 μm capsaicin caused a rapid and reversible increase in afferent discharge. *a*, nerve discharge rate. *b*, raw recording of whole nerve activity in a 3‐month preparation. *B*, compared to 3‐month samples (*n* = 11), the afferent discharge profile in response to bath application of 1 μm capsaicin was significantly attenuated in both 12‐month (*n* = 5) and 24‐month samples (*n* = 9). Significant differences were observed in the 24‐month preparations at 35 s (^*^ *P* < 0.05). *C*, single unit analysis of the multiunit responses revealed that the 24‐month colonic afferents responded to capsaicin (*n* = 9) with an attenuated response profile compared to the 3‐month samples (*n* = 8). Significant differences were observed at 30 s (^*^ *P* < 0.05). Twelve‐month preparations (*n* = 6) possessed similar single unit responses to capsaicin as 3‐month samples. *D*, 24‐month HT afferents (*n* = 5) responded to bath application of 1 μm capsaicin with a diminished response profile compared to 3‐month HT units. Significant differences were observed at 30 and 40 s (^**^ *P* < 0.01, ^***^ *P* < 0.001).

Brief exposure of colonic preparations to the TRPV1 receptor agonist capsaicin (3 ml bath perfusion of 1 μm capsaicin) induced a rapid and reversible increase in afferent discharge following a short latency period (Fig. 3 *A*). These experiments were performed after the mechanosensitive experiments in order to minimise the risk of capsaicin‐induced desensitisation of mechanosensitive afferents, as has been previously described (Rong *et al*. 2004; Brierley *et al*. 2005). Multiunit afferent response profiles to capsaicin were significantly attenuated at 12 and 24 months compared to 3 months (Fig. 3 *B*) (*F* = 8.7, *P* = 0.003 and *F* = 10.97, *P* = 0.001, respectively), although single unit analysis revealed that only the 24‐month afferent response profiles were significantly decreased compared to 3‐month controls (Fig. 3 *C*) (*F* = 8.54, *P* = 0.0039) whereas the 12‐month samples were unchanged from 3 months (*F* = 1.16, *P* = 0.28).

We also analysed the HT mechanosensitive afferent response to capsaicin at 3 and 24 months. In these experiments we found that similar proportions of 3‐ and 24‐month HT afferent fibres responded to capsaicin (100% of 3‐month (6 out of 6 afferents) and 62.5% of 24‐month (5 out of 8 afferents), *P* > 0.05, Fisher's exact test), but that the capsaicin‐induced increase in afferent discharge in 24‐month HT afferents was attenuated compared to that observed in 3‐month samples (Fig. 3 *D*) (*F* = 5.2, *P* < 0.05).

### Expression of TRPV1 and 5‐HT_3A/B_ mRNA in DRG neurons is unaltered with age

**Figure 4:**
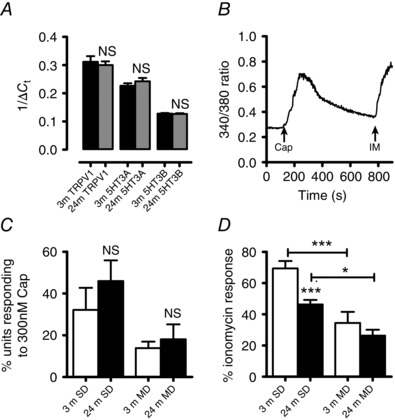
**Age‐related decrease in TRPV1 sensitivity to capsaicin in dorsal root ganglion neurons** Messenger RNA (mRNA) expression for TRPV1 and 5‐HT_3A/B_ receptor subunits in 3‐month (*n* = 3) and 24‐month (*n* = 4) DRG neurons relative to the housekeeping gene GAPDH. *A*, no significant change in target mRNA expression was seen in 24‐month (24 m) DRG neurons relative to 3‐months (3 m). *B*, the time course of an intracellular calcium signal induced in a single 3‐month‐old small diameter (SD) DRG neuron by the addition of 300 nm capsaicin (Cap) followed by addition of 5 μm ionomycin (IM). *C*, the percentage of SD and medium diameter (MD) DRGs that respond to capsaicin was unchanged by age. *D*, bath addition of 300 nm capsaicin gave rise to attenuated responses in 24‐month SD DRGs compared to 3 months. SD DRGs from both 3‐ and 24‐month samples possessed an augmented response to capsaicin compared to MD DRGs. ^*^ *P* < 0.05, *t* test; ^***^ *P* <  0.0001, one‐way ANOVA with Tukey's *post hoc* test.

TRPV1 and 5‐HT_3A/B_ transcripts were detected by qRT‐PCR in 3‐ and 24‐month DRGs (Fig. 4 *A*), but there was no significant age‐related change in their mRNA expression levels (Fig. 4 *A*). However, the relative expression of 5‐HT_3A_ : 5‐HT_3B_ mRNA was greater in both 3‐ and 24‐month DRGs (*P* < 0.0001 for both 3‐ and 24‐month, one‐way ANOVA with Tukey's *post hoc* test).

### TRPV1‐mediated responses are attenuated in aged DRGs

The effects of capsaicin on mobilising [Ca^2+^]_i_ were examined on small diameter (SD, mean diameter of 21.7 ± 0.36 μm (*n* = 48) and 21.4 ± 0.39 μm (*n* = 39) for 3‐ and 24‐month animals, respectively) and medium diameter DRGs (MD, mean diameter of 31.9 ± 0.82 μm (*n* = 48) and 32.14 ± 0.74 μm (*n* = 44) for 3‐ and 24‐month animals, respectively). Capsaicin (300 nm) evoked increases in [Ca^2+^]_i_ in SD and MD DRGs which were rapid in onset and reached a plateau approximately 30 s after exposure (Fig. 4 *B*). There was no significant change in the percentage of SD and MD DRGs that responded to capsaicin at either 3 or 24 months (Fig. 4 *C*). However, the capsaicin‐induced increase in [Ca^2+^]_i_ was attenuated in SD DRGs at 24 months whereas the MD DRG response to capsaicin was unaltered between 3 and 24 months of age (Fig. 4 *D*). In addition, 3‐ and 24‐month SD DRGs gave an augmented response to capsaicin compared to their aged equivalent MD DRGs (Fig. 4 *D*).

### Colonic distribution of enterochromaffin cells in 3‐ and 24‐month animals

**Figure 5:**
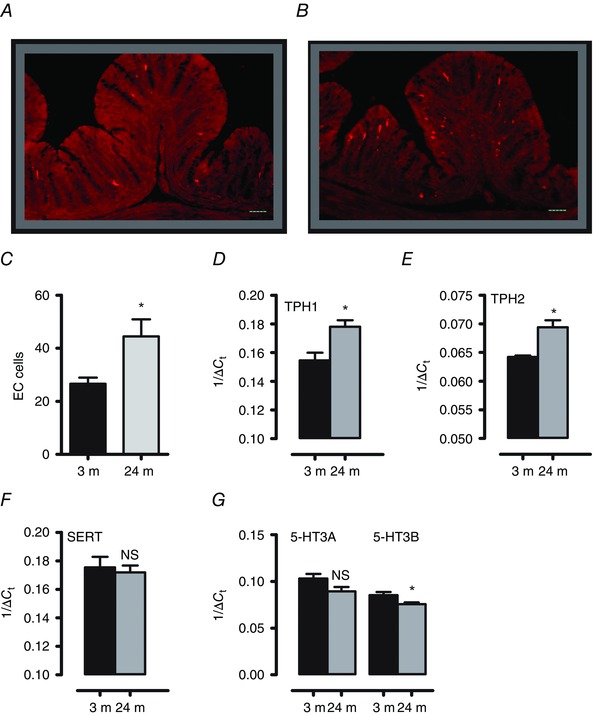
**Reduced components of the 5‐HT signalling pathways in the colon of aged mice** *A* and *B*, images representing examples of 5‐HT immunoreactive EC cells in a cross section of the distal colon in 3‐month (*A*) and 24‐month mice (*B*). Scale bar = 50 μm. *C*, the number of EC cells was significantly increased in the 24‐month colons (*n* = 8 animals) compared to 3 months (*n* = 6 animals). *D–G*, messenger RNA (mRNA) expression for TPH1, TPH2, SERT and 5‐HT_3A/B_ receptor subunits in 3‐month (*n* = 4) and 24‐month (*n* > 6) distal colon segments relative to the housekeeping gene GAPDH. Both TPH1 and TPH2 mRNA transcripts were increased at 24 months compared to 3 months, whereas 5‐HT_3B_ mRNA transcripts were decreased. SERT and 5‐HT_3A_ mRNA levels were unchanged between 3 and 24 months (^*^ *P* < 0.05, unpaired *t* test).

EC cells were clearly identified in the colonic mucosa using a serotonin antibody staining protocol (Fig. 5 *A* and *B*). We found an increased number of EC cells in the 24‐month colonic preparations compared to 3 months (Fig. 5 *C*).

### Colonic expression of components of the serotonergic signalling pathway in 3‐ and 24‐month animals

qRT‐PCR was used to assess the expression levels of TPH1, TPH2, SERT and 5‐HT_3A/B_ transcripts in distal colonic segments. Transcripts for all genes were detected in both 3‐ and 24‐month animals (Fig. 5 *D–G*), but TPH1 and TPH2 expression levels were significantly increased in 24‐month samples compared to 3 months, whereas SERT transcript expression was unaltered by age (Fig. 5 *D–F*). 5‐HT_3B_ expression was significantly decreased in 24‐month samples, while 5‐HT_3A_ transcript levels were unchanged.

### The response of colonic afferents to bath application of the 5‐HT is altered in aged animals

**Figure 6:**
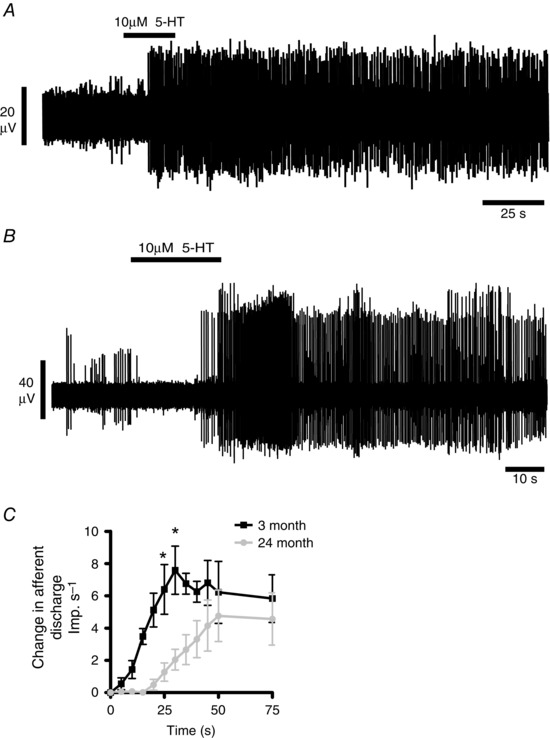
**The response of colonic splanchnic afferent nerves to serotonin is reduced in the colon of aged mice** *A* and *B*, bath perfusion of 10 μm 5‐HT caused an increase in afferent discharge in both 3 month (A) and 24 month (B) animals. *C*, the group afferent response profile to bath application of 10 μm 5‐HT was significantly decreased in 24‐month animals (*n* = 12) compared to 3‐month animals (*n* = 6). Significant differences were observed at 25 and 30 s (^*^ *P* < 0.05).

In 3‐month animals (*n* = 6) brief exposure of colonic preparations to serotonin (3 ml bath perfusion of 10 μm serotonin) induced a rapid and reversible increase in afferent discharge following a short latency period (Fig. 6 *A*). In 24‐month samples (*n* = 12) both the latency and the afferent response profile to serotonin were significantly attenuated compared to 3 months (Fig. 6 *B* and *C*) (*F* = 46.06, *P* < 0.0001).

### Age‐related changes in baseline jejunal afferent activity

Baseline jejunal afferent discharge rates were also significantly attenuated in 24 month animals compared to 3 month (27.2 ± 3.8 imp s^−1^ *versus* 15.0 ± 1.8 imp s^−1^ respectively, *P* < 0.05, Dunnett's *post hoc* test), whereas baseline activity at 12 month (27.5 ± 3.5 imp s^−1^) was indistinguishable from 3 month values (*P* > 0.05 *versus* 3 months, Dunnett's *post hoc* test).

### Age‐related changes in jejunal afferent mechanosensitivity

**Figure 7:**
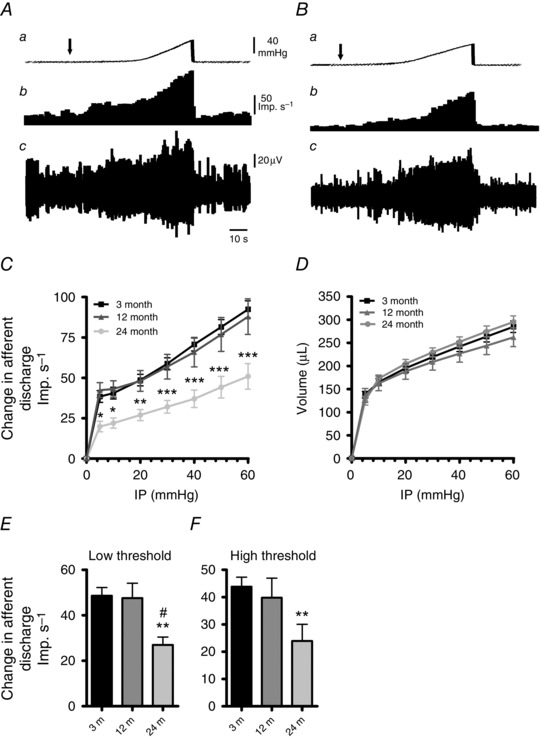
**Ageing differentially affects jejunal afferent mechanosensory function** *A* and *B*, representative traces of the jejunal mesenteric afferent nerve response to ramp distension in 3‐month (*A*) and 24‐month (*B*) animals. *Aa* and *Ba*, ramp distension profile. Arrow denotes start of ramp distension. *Ab* and *Bb*, nerve discharge rate. Note the depressed low‐threshold response in the aged example. *Ac* and *Bc*, raw recording of whole nerve activity. *C*, pressure–response profiles of multiunit activity from 3‐month (*n* = 22), 12‐month (*n* = 11) and 24‐month (*n* = 12) preparations. Pressure–response profiles were unchanged at 12 months but were attenuated at 24 months compared to 3‐month controls. Significant differences were detected above 5 mmHg at 24 months (^*^ *P* < 0.05, ^**^ *P* < 0.01, ^***^ *P* < 0.0001). *D*, compliance curves for the three age groups. Compliance curves were unaltered for both 12‐ and 24‐month preparations compared to 3‐month samples. *E* and *F*, comparison of the afferent discharge responses at low‐threshold (0–20 mmHg; *E*) and high‐threshold (20–60 mmHg; *F*) distension pressures. Both low‐threshold and high‐threshold responses are attenuated at 24 months, whilst the responses at 12 months remain unchanged (^#^ *P* < 0.05 and ^**^ *P* < 0.01, one‐way ANOVA with Tukey's *post hoc* analysis for 12 months *versus* 24 months and 3 months *versus* 24 months, respectively).

The effect of age upon jejunal afferent nerve mechanosensitivity was investigated in 3‐, 12‐ and 24‐month preparations using a repeated ramp distension protocol. In all age groups, ramp distensions induced a robust biphasic increase in afferent discharge in response to ramp distensions (Fig 7 *A* and *B*). The 24‐month jejunal afferent response profile to ramp distension was attenuated compared to the 3‐month samples, whereas the 12‐month afferent response profile was unchanged from 3 months (*F* = 126.85, *P* < 0.0001 and *F* = 0.2, *P* = 0.65, respectively). These changes in afferent signalling occurred in the absence of changes in compliance between the three age groups tested (Fig. 7 *D*) (*F* = 2.45, *P* = 0.1 and *F* = 0.34, *P* = 0.2 for 12 and 24 months *versus* 3 months, respectively). We summarised these results in a series of bar graphs to illustrate the change in afferent discharge at low‐threshold and high‐threshold distension pressures. Low‐ and high‐threshold responses were significantly attenuated in only the 24‐month animals compared to 3‐month controls (Fig. 7 *E* and *F*).

## Discussion

This study demonstrated that sensory signalling is attenuated in the aged murine gastrointestinal tract, affecting both the small and large intestine. We found that the spontaneous discharge of gastrointestinal afferent fibres and their mechanosensory function was attenuated with age, and that colonic high‐threshold mechanosensory fibres were most susceptible to the effects of ageing. These afferent fibres play an important role in transducing nociceptive information to the CNS, but they also have the capacity to sensitise in response to injurious situations, and their loss of function with age may predispose the elderly towards lower awareness of GI injury or disease. Little is known about the effects of age upon gastrointestinal sensory physiology, although clinical studies have demonstrated diminished sensory function in both the upper and lower GI tract (Lasch *et al*. 1997, 1999; Gururatsakul *et al*. 2010).

Our experiments on the ageing mouse showed that mechanosensitivity was attenuated in the aged colon. These effects became apparent at 12 months, were further decreased by 24 months, and were associated with diminished mechanosensory afferent function affecting high‐threshold afferent fibres most potently. We saw no age‐related change in the mechanosensitive activity of wide dynamic range and low‐threshold mechanosensory colonic afferents. This suggests that high‐threshold afferent fibres are most susceptible to age‐related loss of function, and that age‐related changes in mechanosensitivity appear approximately in midlife. Similar to other studies, we found no significant evidence of age‐related neuronal loss (Bergman & Ulfhake, 1998; Phillips & Powley, 2007; Phillips *et al*. 2010), and in a series of pilot studies we found that ageing had no effect upon the electrical properties of these neurons in terms of their resting or threshold potentials (data not shown). Consequently the changes we observed most probably reflected altered mechanosensing properties of these neurons, with at least some of the changes occurring at the level of the nerve terminals within the gut wall. Previous studies on extrinsic afferents innervating the gut have highlighted age‐related changes in the physiology of afferent terminals in the muscle wall and mucosa (Phillips & Powley, 2007; Phillips *et al*. 2009).

Our study used an integrated stimulus approach to activate mechanosensitive afferents by distending segments of gut and recording the afferent traffic that resulted from this stimulus. This protocol provides a way of recording the response to a physiological manipulation of the gut, and also takes into account an integrated response of the muscle layers to distension, but unlike the flat sheet colonic afferent recording techniques (Brierley *et al*. 2004), we cannot identify the precise location of splanchnic afferent nerve endings within the gut wall (mucosal, muscular, mesenteric and serosal). High‐threshold splanchnic afferents comprise the mesenteric and serosal afferents (Brierley *et al*. 2004), which can be activated at nociceptive levels of distension that were reached in our experiments (Brierley *et al*. 2008). We identified high‐threshold afferents in all three age groups studied that exhibited firing characteristics of both serosal and mesenteric afferents, but while we could not determine whether ageing preferentially affected one high‐threshold subtype over another, our data conclusively showed that ageing blunted high‐threshold mechanosensory afferent activity.

Although we saw no changes in low‐threshold mechanosensory function in this study, we cannot be sure that low‐threshold colonic mechanosensory function is preserved with age, since we did not make any recordings from the pelvic afferents, which are more tuned to signal physiologically based stimuli (Brierley *et al*. 2004). It is conceivable that pelvic neurons also exhibit an age‐related loss of mechanosensitivity, which may well impact upon low‐threshold afferent fibres more fully than is seen in the splanchnic neurons.

Information regarding the cellular changes that ageing afferents undergo is limited, although it is clear that afferent function is dependent upon the expression of ion channels and receptors on their terminals that respond to selective stimuli. One family of receptors involved in these processes is the transient receptor potential receptors including the TRPV1 channel (Caterina *et al*. 1997). TRPV1 is well established as playing a key role in detecting potentially injurious events occurring in neurons (Tominaga & Julius, 2000) such as inflammatory and neuropathically invoked nociception. Studies in which TRPV1 activation has been targeted, either through genetic deletion of the receptor or by pharmacological blockade of the channel, have been shown to alleviate thermal hyperalgesia and mechanical allodynia derived from inflammation or nerve injury (Walker *et al*. 2003; Kasama *et al*. 2007). TRPV1 channel activity in the gut is similarly well documented. The receptor has a high expression in neurons innervating the gut (Christianson *et al*. 2006), and TRPV1 knockout mice exhibit loss of visceral afferent mechanosensitivity (Rong *et al*. 2004; Jones *et al*. 2005; Bielefeldt & Davis, 2008). Therefore TRPV1 has an important role in transducing mechanosensitivity within the gut, but is also upregulated by inflammation and contributes towards the generation of visceral hypersensitivity (Miranda *et al*. 2007; Winston *et al*. 2007; Akbar *et al*. 2008). Loss of TRPV1 function with age could therefore impact upon mechanosensing and the ability of selective afferents to sensitise in response to inflammation.

Our experiments on the ageing mouse examined the effects of TRPV1 activation upon splanchnic afferent discharge. Capsaicin‐evoked discharge of single unit colonic afferents was preserved at 12 months, but attenuated in 24‐month samples, suggesting that an age‐related loss in TRPV1 channel function was occurring from midlife onwards. Focusing our analysis upon the aged mouse ganglia and extrinsic nerves suggested that TRPV1 activity is diminished on specific populations of neurons. At 24 months, TRPV1 activity on high‐threshold mechanosensitive afferents was attenuated, and capsaicin evoked calcium signals in small diameter DRG neurons, which have been shown to be the predominant type that innervates the distal colon (Hicks *et al*. 2002), were similarly diminished whilst medium diameter DRG responses to capsaicin remained unaffected. Simultaneously, ageing had no effect upon the number of capsaicin‐sensitive units, and nor did we detect changes in TRPV1 mRNA expression in pooled DRGs from 3‐ and 24‐month animals. This suggested that any age‐related changes in TRPV1 channel function were likely to be at the translational level. Interestingly this observation was made in cutaneous sensory neurons in which reduced TRPV1 protein was observed in aged sensory neurons (Wang *et al*. 2006).

It must be noted, though, that we did not use retrograde labelling to identify specific gut innervating DRGs during these experiments, and as such it was likely that some of the DRGs examined for both molecular and electrophysiological studies innervated other visceral regions. However, the loss of TRPV1 function on the HT afferents may well underlie the blunted mechanosensory response in these afferents, but is also likely to impact upon their ability to sensitise in response to inflammation.

Due to the divergent neuroprotective and proinflammatory roles that 5‐HT plays within the gut (Bischoff *et al*. 2009; Ghia *et al*. 2009; Li *et al*. 2011; Gershon, 2012), we also investigated age‐associated effects upon serotonergic signalling pathways. At 24 months, we found increased EC cell numbers and TPH1 mRNA expression in colonic samples compared to 3‐month animals. This supported previous studies highlighting elevated serotonin‐immunoreactive cells in the stomach and small intestine of aged rodents (Khomeriki, 1989; Sandstrom & El‐Salhy, 2000). We also detected elevated expression of TPH2 mRNA in the 24 month colon, suggesting an age‐related increase in neuronal 5‐HT depots. This suggests that there is elevated 5‐HT in the aged gut. However, compared to 3‐month samples, 5‐HT‐invoked sensory signalling processes were attenuated at 24 months, affecting both the latency and the afferent response profile to bath applied 5‐HT. This would seem counterintuitive given that elevated 5‐HT would likely give rise to enhanced afferent discharge, but elevated gastrointestinal 5‐HT bioavailability, as is seen for example in animals in which the serotonin transporter protein has been deleted, can be associated with changes in 5‐HT_3_ receptor subunit structure (Liu *et al*. 2002), which may impact upon 5‐HT‐evoked afferent signalling processes. However since these experiments only covered two age groups, we need to address the possibility that we were measuring linear changes in gastrointestinal function as opposed to age‐related changes which may develop abruptly at specific time points during healthy ageing.

Our studies also showed that ageing attenuated mechanosensory afferent signalling in the jejunum. We noted that both the low‐ and high‐threshold distension response profiles were attenuated with age, but unlike the colon, these changes were not seen until 24 months of age. We do not know why age‐related changes in sensory function in the small intestine appear to be delayed compared to the colon, although these results are not unique in that morphological studies have demonstrated that age‐related loss of myenteric neurons increased in severity from an oral to anal direction (Phillips and Powley, 2001). It is tempting to speculate that this is a protective mechanism to preserve small intestinal function and its associated nutrient assimilation processes, or that age‐related changes in the colon reflect a lifelong exposure to the microbiome (Lakshminarayanan *et al*. 2014).

Our main experiments used three age groups, which allowed us to study age‐related changes in gastrointestinal sensory function. However, using multiple age groupings in addition to our chosen pools would have allowed us to extrapolate more information regarding subtle changes that may have occurred throughout the lifespan of this animal. Our choice of 3‐month animals as the starting ‘adult’ age group was based primarily upon previous afferent recordings from our group which utilised control mice aged between 3 and 6 months, and in which no significant changes were observed in their afferent mechanosensory or chemosensory responses (Rong *et al*. 2004; Keating *et al*. 2008). Therefore we considered 3 months to be a suitable baseline age in which to start investigating age‐related sensory dysfunction, although prudence would suggest using 6‐ or 8‐month animals in addition to 3‐month animals in future studies. Conversely, the use of a single age grouping to represent ‘old’ held implicit risks: were we looking at ‘sick’ mice or ‘old’ mice? Very old animals are likely to have diseases, and although no autopsies were performed on these animals, we did undertake visual examinations to look for any obvious lesions and abnormalities, and our aged colonies were carefully monitored for signs of ill health. Therefore the three age groups we experimented on were, as far as we could tell, healthy, but future studies would benefit from a small cohort of animals from each age group studied being autopsied. Finally it is worth noting that we used the mechanosensory response to ramp distensions of intestinal tissue as an internal control to test the viability of our tissue over the course of an experiment, as well as a mechanism to investigate mechanosensitivity. Throughout this study, we found that distensions of jejunal and colonic tissue were consistent and reproducible between age groups and that the length of time we could perform physiological experiments before we lost viability of our tissues were no different between the three age groups tested.

In conclusion, the prime finding in this study was the age‐associated attenuation in HT afferent mechanosensitivity affecting the murine colon, and the associated loss of TRPV1 channel function. These units have the capacity to sensitise in response to injurious events, and their loss may predispose the elderly to lower awareness of GI injury or disease, which could impact upon increased health care costs, morbidity and mortality in the ageing population. More studies are therefore required to investigate the mechanisms contributing towards the age‐associated blunting of sensory perception, particularly in the context of visceral inflammation.

## Additional information

### Competing interests

There are no competing interests from any of the authors.

### Author contributions

All experiments were performed in D.G.’s laboratory at the University of Sheffield. C.K. and D.G. were involved in the conception of the study. C.K., L.N., Y.Y., J.D. and D.G. were responsible for the conception and design of the experiments. C.K., L.N., Y.Y. and J.D. were responsible for the collection, analysis and interpretation of data. All authors contributed to drafting sections of the manuscript, and C.K. wrote the MS. All authors were involved in revising it critically for important intellectual content prior to its submission. All authors have approved the final version of the manuscript and agree to be accountable for all aspects of the work. All persons designated as authors qualify for authorship, and all those who qualify for authorship are listed.
